# Medical education research: the application of robust statistical methods

**DOI:** 10.5116/ijme.5181.5fe8

**Published:** 2013-05-08

**Authors:** Mohsen Tavakol, Rand R. Wilcox

**Affiliations:** 1 The University of Nottingham, UK; 2 University of Southern California, USA

In quantitative inquiry approaches, researchers use deductive reasoning in order to formulate their null hypotheses and then they test these hypotheses using a random sample from the target population. Given the sample results, inferences or decisions about the distribution of the population are made. However, if researchers take different samples from the same population, they will obtain different results from each sample. This indicates that the inference about the population based on a random sample may not be correct. Nevertheless, if researchers take many samples from the same population and then calculate the mean for each random sample, the mean of the sample means is equal to the mean of the original population. Moreover, the sampling distribution of the sampling means is approximately normal when the sample size is sufficiently large. Normality of the sampling distribution of the sample mean is a key assumption when using parametric tests, especially if statistical inferences are drawn from the data. If the assumption of normality is violated, control over the type I error probability can be poor and power can be relatively poor as well. Although conventional wisdom is that normality can be assumed with a sample size of 40 or more, it has been well documented that under general conditions this not the case. The one-sample Student’s T test, for example, can require over 300 observations, particularly when dealing with skewed, heavy-tailed distributions.^1^ Outliers are an integral part of heavy-tailed distributions and can lead to Type I and Type II errors.^2^ Problems with parametric tests have been explained by a group of statisticians.^2^,^3^There is a simple solution to ensure the appropriateness and accuracy of the interpretation of p values, effect sizes and confidence intervals. They are modern or robust statistical methods. These methods are able to solve the issues associated with parametric tests when assumptions are violated.^4^ Robust methods provide better results especially if the data are skewed, either positively or negatively. Despite the fact that these methods are used in other disciplines, they have been rarely used in medical education research. Therefore the purpose of this editorial is to explain the application of robust statistical methods in medical education research as medical educators should take advantage of modern advanced statistical procedures. To obtain a greater understanding of robust methods, readers need to consult the references cited in this editorial.

## Issues of parametric statistical tests for comparing groups

Medical education researchers rarely attend to assumptions of normality, linearity and homoscedasticity in order to run parametric tests. These assumptions should be fulfilled in order to use parametric statistical procedures for comparing groups (e.g. t-test, analysis of variance and regression). But in practice, these assumptions are rarely fulfilled, which can result in poor control over the Type I error probability and poor power.^5^ The P values provided by software programs for quantitative data, for example, SPSS, S-Plus, SAS or R, can be unreliable if the assumptions of normality and homoscedasticity are violated. Indeed, non-significant p-values can become significant p-values when using more modern methods.^6^ Normality of variables is usually screened using either statistical procedures, e.g. skewness, kurtosis and the Kolmogorov-Smirnov test, or graphical methods. Levene’s test is used for assessing homoscedasticity. However, numerous publications have found that this approach is unsatisfactory, roughly because they do not have enough power to detect situations where violating assumptions is a practical concern.^7^ When comparing the mean of two independent groups, some statistical packages perform the Welch test, which does not assume that groups have a common variance. The Welch test can yield more accurate confidence intervals in comparison with Student’s T. However, issues continue in terms of both Type I error probabilities and power. Even small departures from normality can be a source of concern.

## Transformations

If variables are skewed, either positively or negatively, researchers transform data in order to reduce the skewness and the kurtosis. Common strategies are to use a square root or log transformation.^2^ However, often distributions remained skewed with outliers. Also, the interpretation of the transformed data sometimes is difficult. A more effective strategy is to use trimmed means in conjunction with bootstrap methods (see below).Robust statistical methods can overcome the issues of non-normality and heteroscedasticity. In particular, they provide good control over the Type I error probability and accurate confidence intervals for a much broader range of situations and they can help increase power when comparing groups and studying associations. Medical researchers rarely perform power analyses to identify appropriate sample sizes.^5^ Modern insights reveal that if a power analysis is performed assuming normality, much larger sample sizes might actually be needed to deal with violations of assumptions. Robust methods can help to reduce this problem substantially. The importance of robust statistical methods is fully discussed elsewhere.^8^,^9^ Below, we briefly describe some basic robust methods that can be used in medical education research.

## Measures of central tendency and variation

Outliers can distort a sample mean and inflate its standard error (the variation of sample means over many studies), which in turn can mean low power. Therefore, we need measures of central tendency and variation that are not affected by outliers. One strategy is to replace the mean with the median. But a central concern is that often the median does not have satisfactory power, roughly because it trims all but one or two of the values. Imagine the following hypothetical data:3, 3, 3, 4, 4, 6, 6, 6, 7, 25, 43The mean of the data is 10. However, this poorly reflects the typical value due to the values 25 and 43, which distort the mean and render it an undesirable measure of central tendency. The median is 6, which is significantly different from the mean and provides a better reflection of the typical response. But because the median trims all but one or two values, power can be poor unless the number of outliers is fairly large. In order to minimise the influence of outliers, a 20% trimmed mean is recommended, which achieves nearly the same amount of power as the mean when the normality assumption is true.^10^ By following this recommendation, our data set is as follows:3, 4, 4, 6, 6, 6, 7So the trimmed mean is 5.1, which better reflects the typical response compared to the mean, which is 10. It has been well documented that methods based on a trimmed mean can provide an accurate confidence interval, good power and good control over the Type 1 error probability for a broader range of situations compared to Student’s T.^3^ It is stressed, however, that merely trimming observations and applying Student’s T to the remaining data is technically unsound and generally yields highly inaccurate results, even with a very large sample size. A technically sound method is outlined below and other methods are available.^8^,^9^If the smallest 20% of observed data are replaced by the smallest value not trimmed and the largest 20% of observed data are replaced by the largest value not trimmed, and then the mean is calculated, this yields what is called the Winsorised mean. Consider again the above example:3, 3, 3, 4, 4, 6, 6, 6, 7, 25, 43Then 20% trimming means that the two largest values, 25 and 43, and the two smallest values are 3 and 3, would be trimmed. We Winsorise these values as follows:3, 3, 3, 4, 4, 6, 6, 6, 7, 7, 7The 20% Winsorised mean is 5.03. Based upon the Winsorised mean, we are now in a position to calculate a robust measure of dispersion called the Winsorised variance (WV). To compute the WV, simply use the traditional formula for the variance applied to the Winsorised values. The Winsorised variance is 2.89 in contrast to the usual variance, which is 159. Other robust measures of central tendency have been addressed elsewhere.^8^,^9^

## Bootstrap methods

In practice, sometimes the observed data come from an unknown distribution that is not normal. Such data can negatively impact classic tests statistic (for example, the t test), especially when two or more assumptions (for example, normality, linearity and homoscedasticity) are violated simultaneously. A strategy for dealing with this problem is to use a bootstrap technique. There are two basic types: a percentile method and a bootstrap-t, both of which begin by generating many bootstrap samples from the original observed data with replacement. Under normality, control over the Type I error probability is nearly as good as classic methods, and when dealing with non-normal distributions it can be substantially better.^9^

The percentile bootstrap method performs very well when using a 20% trimmed mean. (For means, a bootstrap-t is preferable.) If you wish to compute a 95% confidence interval, simply generate many bootstrap samples, say 5000, and then calculate the 20% trimmed mean for each bootstrap sample. Put these 5000 trimmed means in ascending order, in which case the 0.95 confidence interval corresponds to the middle 95%. Statistical software packages can easily apply bootstrap techniques.

## Robust Cronbach’s alpha

Calculating alpha has become popular in medical education research as a measure of reliability. The importance and application of Cronbach’s alpha has been explained elsewhere.^11^ When a test is supposed to be a measure of reliability, it should be reliable for the bulk of participants. Traditionally, we use the individual item variances and total variance in order to calculate the Cronbach’s alpha based on a set of test items. But this method provides a non-robust estimate of test reliability.^12^ Studies have shown that psychometric measures often have a strong-skewed distribution with heavy tails. Even a small departure from normality can substantially influence the variance, which in turn can distort a measure of reliability. Therefore, we need to develop a version of alpha which is resistant to extreme values (outliers).There are a variety of methods for estimating a robust alpha.^12^,^13^ These methods are able to resist extreme values and therefore measures the internal consistency of the middle part of the observed values.^12^ Bootstrapping methods are able to estimate confidence intervals for the alpha. This is very encouraging, especially if data are asymmetrical. Readers who are interested in estimating a robust version of Cronbach’s alpha can consult the papers and books cited in this editorial.

## Conclusions

There are many advances relevant to basic techniques that are impossible to describe here. Robust versions of all the usual ANOVA and ANCOVA methods are now available as well as important improvements relevant to classic nonparametric (rank-based) techniques. Generally, the more complicated the design, the more beneficial are modern methods. Regarding regression, substantially improved techniques for dealing with non-normality, outliers, heteroscedasticity and curvature are now available that often make a substantial difference. Moreover, software for applying these more modern methods is available.^8^,^9^ Finally, modern methods do more than provide good control over theprobability of a Type I error and improved power when standard assumptions are violated. They help provide a deeper and more accurate understanding of what data are trying to tell us. All that remains is taking advantage of what modern technology has to offer.

### Conflict of Interest

The authors declare that they have no conflict of interest.
